# Digital financial inclusion and comprehensive multilevel medical insurance system in China

**DOI:** 10.3389/fpubh.2025.1586780

**Published:** 2025-04-24

**Authors:** Zhe Liu, Xingyuan Wu, Xi Chen, Congzhi Qin, Zenghui Qiu, Zhuangzhi Fang, Lan Yao

**Affiliations:** ^1^School of Medicine and Health Management, Tongji, Medical College, Huazhong University of Science and Technology, Wuhan, China; ^2^School of Economics, Huazhong University of Science and Technology, Wuhan, China; ^3^School of Electronic Information and Communications, Huazhong University of Science and Technology, Wuhan, China; ^4^School of Artificial Intelligence and Automation, Huazhong University of Science and Technology, Wuhan, China

**Keywords:** digital financial inclusion, multilevel medical insurance system, panel data regression, causal forest model, double machine learning

## Abstract

**Background:**

The influence of digital financial inclusion on medical insurance systems has drawn significant attention. However, its impact on China’s multilevel medical insurance system (MMIS) remains uncertain. The MMIS is crucial for societal wellbeing. It safeguards citizens from financial hardships during medical treatment, especially as the population ages and healthcare costs rise. By covering medical expenses, the MMIS ensures equal access to essential medical services, protecting people’s health rights and preventing impoverishment caused by medical expenses, thus promoting social stability and equity. This study used panel data from 31 provinces in China from 2016 to 2020 to explore the impact of digital financial inclusion on the development of the MMIS.

**Methods:**

An MMIS development matrix was constructed using the entropy weight method and the Technique for Order Preference by Similarity to an Ideal Solution (TOPSIS). Panel data regression models were employed to analyze the effects of different dimensions of digital financial inclusion on the MMIS.

**Results:**

The overall digital financial inclusion index, particularly the “coverage” dimension, had a significant positive and significant impact on the MMIS (*p* < 0.05). However, the “depth” and “digitization” dimensions were statistically insignificant (*p* > 0.1). Robustness checks using lagged instrumental variable approaches and double machine learning (DML) methods confirmed the stability of these findings. Heterogeneity analysis using the causal forest model revealed that digital financial inclusion had a more pronounced effect in central and western regions. Its impact in the eastern regions was relatively weaker, highlighting significant regional disparities.

**Conclusion:**

Digital infrastructure in the central and western regions must be strengthened to enhance financial inclusion coverage and service capacity; the depth and quality of financial services in the eastern regions should be improved to optimize the effects of digital finance on the MMIS. Deeper integration between digital financial inclusion and the MMIS should be prompted through technological innovations to improve both equity and sustainability. This study provides valuable insights for policymakers to address regional disparities in further developing the MMIS.

## 1 Background and research hypotheses

### 1.1 Background

By implementing policies and measures to encourage healthy behaviors and improve access to quality healthcare, the “Healthy China” initiative seeks to build a sustainable health system and contribute to the overall wellbeing and productivity of the nation (1). A multilevel medical insurance system (MMIS) is fundamental to achieving the strategic goals of this initiative. It is also a key policy measure for addressing the challenges posed by an aging society (2), safeguarding citizens’ health rights, and preventing impoverishment due to catastrophic medical expenditures. Over the past decade, China has made significant progress in developing an MMIS centered on basic medical insurance (3), which includes critical illness insurance, medical assistance, and commercial health insurance. However, the system continues to face considerable challenges owing to disparities in residents’ financial capacity (4), limited coverage, and the substantial healthcare burden on certain population groups. Consequently, effective policy interventions are urgently required to enhance resource allocation and promote greater equity and sustainability within the existing system (5).

Digital inclusive finance, characterized by its low cost, wide accessibility, and operational convenience, is a promising solution for optimizing the MMIS. By leveraging digital technology and integrating financial services, digital inclusive finance can effectively lower the costs associated with insurance enrollment, increase participation rates, and utilize advanced data technologies for precise risk assessments (6). Consequently, tailored insurance schemes addressing the diverse needs of different income groups can be provided. Moreover, digital inclusive finance can facilitate innovation in medical insurance management, improving the overall operational efficiency and governance of the MMIS.

Existing research on the impact of digital inclusive finance on medical insurance systems has largely focused on the experiences of different countries and its applications within diverse economic contexts. However, the impact of digital inclusive finance on China’s MMIS has yet to be fully investigated (7). Furthermore, these studies offer limited insights into the specific mechanisms and pathways through which digital inclusive finance influences healthcare coverage and equity. Therefore, despite the rapid advancement of digital inclusive finance in China, its precise role and region-specific effects remain unclear, particularly in underdeveloped regions. This study addresses this research gap by proposing a provincial MMIS development index, constructed using provincial-level healthcare data from 2016 to 2022, and employing the Peking University Digital Inclusive Finance Index to evaluate the impact of digital inclusive finance on the MMIS and investigate its mechanisms in underdeveloped regions (8). The findings are expected to contribute to a deeper understanding of the role of digital inclusive finance in strengthening China’s MMIS and to provide policy recommendations for further optimization.

### 1.2 Research hypotheses

Based on the multidimensional framework of digital financial inclusion (DFI), this study proposes that its overall index and sub-dimensions positively influence the MMIS. The hypotheses and supporting evidence are as follows:

H1: The overall index of digital financial inclusion positively enhances MMIS.

**Mechanism**: DFI integrates payment, credit, and insurance services, lowering barriers to healthcare access (e.g., rural residents, gig workers). For example, online platforms like Alipay’s “Citizen Services” simplify insurance enrollment, reducing coverage gaps. Commercial health insurance supported by DFI supplements basic medical insurance (e.g., critical illness coverage), improving systemic resilience (9).

H2: The coverage dimension of DFI positively impacts MMIS.

**Mechanism**: Mobile payment and digital accounts expand healthcare access to remote areas. For instance, agent banking networks enable rural populations to enroll in insurance systems, while digital ID verification allows migrant workers to bypass residency restrictions (10).

H3: The depth dimension of DFI strengthens MMIS.

**Mechanism**: Tailored financial products (e.g., medical installment loans, blockchain-based insurance claims) alleviate sudden medical expenses. For example, low-income patients use DFI-enabled microloans to prepay surgery costs, reducing poverty risks (11).

H4: The digitization dimension of DFI drives MMIS.

**Mechanism**: AI and big data optimize healthcare resource allocation. For instance, credit scoring models identify high-risk populations for preventive insurance, while smart contracts automate claims processing, reducing delays.

While Hypothesis H1 posits a positive effect of overall digital financial inclusion (DFI) on the MMIS, structural factors such as residents’ financial capacity, population scale, regional economic strength, and openness—captured by control variables including per capita disposable income, total population, regional GDP, and degree of openness (Section 2.2.3)—may moderate this relationship. For example, in regions with lower per capita income, the impact of DFI on MMIS may be stronger as digital services fill gaps in traditional financial access, whereas in high-income areas, established financial systems might reduce the marginal benefit of DFI. Large populations could challenge the uniform delivery of digital services, potentially weakening DFI’s effect, while high openness might expose regions to international financial risks that influence DFI’s stability in supporting MMIS.

These contextual disparities motivate the heterogeneity analysis in Section 3.3, where we introduce interaction terms between the DFI total index and structural variables (per capita disposable income, total population, regional GDP, and degree of openness) to examine how economic and demographic conditions shape DFI’s impact. Additionally, regional heterogeneity is explored by dividing provinces into eastern, central, and western groups, reflecting varying levels of economic development and digital readiness that may amplify or attenuate the overall effect of DFI on MMIS. By focusing on the total DFI index—consistent with our empirical design—we investigate whether its benefits for MMIS are uniform across contexts or contingent on local conditions.

## 2 Methods and data sources

### 2.1 Data sources

The study utilizes four primary data sources, each described in detail below to ensure transparency and replicability:

#### 2.1.1 China health statistics yearbook (2016–2020)

- **Publisher:** National Health Commission of the People’s Republic of China; published annually by China Statistics Press.
- **Content:** Provides standardized province-level statistics on healthcare financing and insurance coverage. Variables derived from this source inform the construction of the MMIS development matrix, including per capita basic medical insurance fund expenditure, fund coverage of medical revenue, economic security ratio of total health expenditure, average benefit frequency for employee and resident populations, and per capita subsidies for cooperative medical schemes.
- **Access:** Publicly available through official government releases (e.g., 2020 Yearbook PDF) and via academic databases such as CNKI and Wanfang Data.
- **Processing:** After extraction from relevant chapters (e.g., “Medical Insurance”), missing values were imputed using mean substitution. All variables were then standardized using min–max normalization to ensure comparability across provinces and years.

#### 2.1.2 China insurance yearbook (2016–2020)

- **Publisher:** China Insurance Regulatory Commission; published annually by China Insurance Yearbook Publishing House.
- **Content:** Contains province-level data on the development of commercial health insurance, including health insurance premium income and claim payouts, which serve as key inputs for assessing the supplementary insurance component of the MMIS framework.
- **Access:** Accessed via academic databases (e.g., CNKI, Wanfang Data) and yearbook collection websites such as the Statistical Yearbook Download Site.
- **Processing:** The claim ratio was calculated as the ratio of total claims to premium income. Missing values were first imputed using mean substitution, followed by min–max normalization to standardize variables across provinces and years.

#### 2.1.3 Peking University digital financial inclusion index (PKU-DFIIC, 2016–2020)

- **Developer**: Peking University Digital Finance Research Center in collaboration with Ant Financial Services Group.
- **Content**: The core independent variable, the Digital Financial Inclusion Index (DFI), and its three sub-indices (coverage, depth, digitization). The index is constructed using big data from Alipay transactions, mobile payment records, and financial service usage metrics, providing granular provincial-level measurements of digital financial development.
- **Access**: Institute of Digital Finance, Peking University^1^ publicly releases reports and raw index data.
- **Processing**: The raw index values were logarithmically transformed (ln_dif) to reduce skewness, as recommended by the index developers to enhance statistical modeling stability.

#### 2.1.4 China economic and social big data research platform (CNKI)

- **Platform Operator:** China National Knowledge Infrastructure (CNKI), a leading academic database aggregator in China.
- **Content:** Serves as the primary portal for accessing standardized macroeconomic and social statistics, integrating data from authoritative sources such as the National Bureau of Statistics, Ministry of Industry and Information Technology, and provincial statistical bureaus.
- **Access:** Accessed via institutional subscriptions (e.g., university libraries) through the CNKI portal,^2^ which provides structured datasets.
- **Processing:** Data were extracted using CNKI’s China Economic and Social Big Data Research Platform, ensuring consistency in variable definitions (e.g., GDP at current prices). Control variables were calculated as specified in Section 2.2 (e.g., industrial upgrading as tertiary industry value added/secondary industry value added).

#### 2.1.5 Data integration and consistency checks

- **Time Period**: All datasets were aligned to the 2016–2020 period, corresponding to the availability of consistent provincial-level MMIS and digital finance data.
- **Geographic Scope**: Covers all 31 provinces in mainland China, excluding Hong Kong, Macau, and Taiwan due to data incomparability.

### 2.2 Variable description

#### 2.2.1 Independent variable: digital financial inclusion

The Digital Financial Inclusion Index (DFI) of China, developed by the Peking University Digital Finance Research Center in collaboration with Ant Financial Services Group, serves as the core independent variable. This index measures the penetration and utilization of digital financial services across three sub-dimensions:

- **Coverage**: Reflects the accessibility of digital financial services, operationalized by metrics such as the penetration rate of Alipay accounts, the proportion of users linking bank cards to digital platforms, and the geographic reach of mobile payment networks. This dimension captures the extent to which populations, particularly in remote or underserved areas, have access to basic digital financial tools (e.g., mobile wallets, digital accounts).
- **Depth**: Captures the intensity and diversity of digital financial service usage, including transaction volumes (e.g., online payments, peer-to-peer lending), the variety of financial products utilized (e.g., insurance, wealth management), and the frequency of interactions with digital financial platforms. This sub-index reflects how deeply individuals and businesses integrate digital finance into their economic activities.
- **Digitization**: Focuses on the technological efficiency and convenience of digital financial services, such as the prevalence of mobile-first interfaces, algorithm-driven risk assessment (e.g., credit scoring models), and automation in service delivery (e.g., smart contracts for claims processing). This dimension emphasizes the technological infrastructure enabling seamless, low-cost financial transactions.

**Advantages**: The DFI offers a comprehensive, multi-dimensional measure of digital financial development, leveraging large-scale transaction data to capture real-world usage patterns. Its provincial-level granularity allows for regional comparisons.

**Limitations**: While the index highlights access and usage, it may underrepresent qualitative aspects, such as user trust in digital platforms or the equity of service distribution across socioeconomic groups. Additionally, the reliance on Alipay-based data may slightly bias results toward regions with higher Ant Financial service penetration.

#### 2.2.2 Dependent variable: MMIS

MMIS score, which reflects the maturity and comprehensiveness of provincial health insurance systems in China. The score was constructed using eight indicators, including per capita basic medical insurance fund expenditure, fund coverage of medical revenues, economic protection of total health expenditure, average benefit frequency for employees and residents, per capita government subsidies for cooperative medical schemes, and the commercial health insurance claim ratio. These indicators collectively capture key dimensions of both basic and supplementary health insurance coverage.

All component variables were standardized using min–max normalization prior to aggregation in order to ensure comparability across provinces and reduce the influence of scale differences.

**Advantages:** The MMIS score provides a multi-dimensional measure of health insurance development, integrating public and private sector data to reflect the institutional capacity and structural depth of health financing systems at the provincial level.

**Limitations:** The use of aggregated provincial data may overlook intra-regional disparities and cannot capture individual-level outcomes such as equity of access, quality of service, or user satisfaction.

#### 2.2.3 Control variables

To mitigate potential biases caused by omitted variables, several control variables were included in the analysis, with some variables selected based on existing literature (12). They were listed as follows, each described below with its economic meaning, calculation method, and evaluation of strengths/limitations:

- **Degree of openness**:

o **Meaning**: Measures a region’s economic integration with the global market, reflecting its exposure to international trade.
o **Calculation**: Ratio of total imports and exports to GDP (total trade volume/GDP).
o **Advantages**: Directly quantifies the degree of economic openness, crucial for understanding how global economic linkages may influence local financial and healthcare systems.
o **Limitations**: Focuses on goods trade only, potentially overlooking service trade or capital flows, and may not fully capture the complexity of economic openness (e.g., foreign direct investment).

- **Fiscal scale**:

o **Meaning**: This reflects government fiscal capacity and intervention in the economy, indicating the role of public spending in healthcare and social security.
o **Calculation**: Ratio of general public budget expenditure to GDP (government spending/GDP).
o **Advantages**: A standard measure of fiscal policy strength, essential for assessing the government’s ability to fund medical insurance initiatives.
o **Limitations**: Does not account for fiscal efficiency (e.g., how effectively funds are allocated) or debt sustainability, which may impact long-term MMIS sustainability.

- **Industrial upgrading**:

o **Meaning**: Captures the shift toward service-oriented economies, a proxy for economic structural advancement.
o **Calculation**: The ratio of the annual increase in the tertiary industry to the annual increase in the secondary industry. (Annual increase in tertiary GDP/annual increase in secondary GDP).
o **Advantages**: Highlights progress toward post-industrial economic structures, which often correlate with higher productivity and potential for innovative healthcare financing models.
o **Limitations**: Ignores the quality of tertiary industry development (e.g., low-value vs. high-value services) and may not directly reflect regional healthcare service capacity.

- **Per capita disposable income**:

o **Meaning**: Represents residents’ financial capacity and purchasing power, a key determinant of healthcare affordability and insurance participation.
o **Calculation**: Total household disposable income divided by population.
o **Advantages**: A direct measure of residents’ financial wellbeing, critical for understanding demand-side drivers of MMIS participation.
o **Limitations**: Averages may mask income inequality within regions, and non-monetary welfare (e.g., employer-provided insurance) is not captured.

We also considered other variables that may influence the MMIS, as listed below:

- **Dependency ratio**:

o **Meaning**: Measures demographic pressure, defined as the ratio of non-working-age populations (children and older adult) to the working-age population (15–64 years).
o **Calculation**: (Population <15 + Population ≥65) /Population 15–64.
o **Advantages**: Indicates the burden of healthcare and pension costs on the working-age population, relevant for assessing MMIS sustainability.
o **Limitations**: Simplifies demographic complexity by treating children and the older adult as a single group, ignoring differing healthcare needs between the two.

- **Regional GDP**:

o **Meaning**: Reflects a region’s overall economic size and productive capacity, influencing healthcare resource availability.
o **Calculation**: Gross Domestic Product at the provincial level.
o **Advantages**: A comprehensive measure of economic strength, widely used to proxy regional development levels.
o **Limitations**: Does not account for GDP composition (e.g., reliance on extractive industries vs. technology) or environmental costs, which may indirectly affect healthcare burdens.

- **Total population**:

o **Meaning**: Captures regional demographic scale, impacting the demand for and scalability of medical insurance services.
o **Calculation**: Total resident population of each province.
o **Advantages**: Essential for understanding the size of the insured population and potential economies of scale in insurance administration.
o **Limitations**: Does not reflect population density or urban–rural distribution, which significantly affect healthcare access and service delivery costs.

- **Technological innovation**:

o **Meaning**: Measures the capacity for technological advancement and its application in healthcare, such as digital health solutions.
o **Calculation**: Value of technology market transactions, reflecting the commercialization of innovative technologies.
o **Advantages**: Quantifies the regional ability to generate and adopt new technologies, critical for digital healthcare integration.
o **Limitations**: Focuses on transaction volume rather than innovation quality and may overstate impact in regions with large but inefficient technology markets.

- **Network infrastructure**:

o **Meaning**: Represents the availability of digital infrastructure, a prerequisite for accessing digital financial and healthcare services.
o **Calculation**: Number of internet access port users, including broadband and mobile network ports.
o **Advantages**: A tangible measure of digital connectivity, directly influencing the usability of online insurance platforms and data-driven healthcare tools.
o **Limitations**: It does not reflect network speed, reliability, or affordability, which are critical for seamless digital service adoption, especially in rural areas.

### 2.3 Entropy weight and TOPSIS method

This study utilized the entropy-weighted TOPSIS method to develop a provincial-level index evaluating each region’s performance in establishing its MMIS. The entropy weight method assigns weights to each indicator based on the degree of variability, thereby reducing subjectivity in the weighting process. Using these weights, the TOPSIS method calculates each province’s distance to the positive and negative ideal solutions to derive a comprehensive score. This approach provides an objective and quantitative assessment of the relative performance of provincial medical insurance systems in China.

### 2.4 Panel data regression

Based on the research hypotheses, the regression model is specified as


(1) $$I{n_{\mathrm{score}}}_{i,t}=\beta_{0}+\beta_{1}I{n_{dif}}_{i,t}+\beta_{2}\mathrm{contro}l_{i,t}+\mu_{t}+\delta_{i}+\varepsilon_{i,t}\text{,}$$

where the subscript *i* represents the individual province, and subscript *t* represents the year. $\mathrm{In}\_\mathrm{scor}e_{i,t}$ denotes the MMIS score of province *i* in year *t*. $\mathrm{In}\_dif_{i,t}$ is an independent variable representing digital financial inclusion. $\mathrm{contro}l_{i,t}$ accounts for other factors that influence the MMIS. $\mu_{t}$ represents time-fixed effects, $\delta_{i}$ denotes individual province-fixed effects, and $\varepsilon_{i,t}$ is the regression error term.

### 2.5 Double machine learning

DML offers unique advantages over traditional causal inference methods in terms of model estimation and variable selection. Unlike traditional econometric models, DML does not require pre-specifying relationships between variables, thereby reducing specification bias. In China, related research is still in its early stages and is primarily applied in areas such as policy impact estimation and mediation effect analysis. This study employed a widely used method proposed by Chernozhukov (13) to construct the following partially linear model:


(2) $$\mathrm{In}\_\mathrm{socr}e_{\mathrm{it}+1}=\theta_{0}\mathrm{In}\_dif_{\mathrm{it}}+g\left(X_{\mathrm{it}}\right)+U_{\mathrm{it}}\text{,}$$

(3) $$E\left(U_{\mathrm{it}}|\mathrm{In}\_dif_{\mathrm{it}},X_{\mathrm{it}}\right)=0\text{,}$$

Where *i* represents the province and *t* represents the year. In_socre is the logarithmic value of the MMIS score, and In_dif represents the logarithmic value of digital financial inclusion. Theta $\theta_{0}$ is the coefficient of digital financial inclusion. $X_{\mathrm{it}}$ denotes the set of control variables, and the specific functional form $g\left(X_{\mathrm{it}}\right)$ is estimated using machine learning algorithms. $U_{\mathrm{it}}$ represents the error term.

If Equations 2, 3 are estimated directly, the resulting θ may be biased because, in high-dimensional or complex model settings, machine learning models introduce regularization terms for dimensionality reduction. Although regularization prevents the variance of estimators from becoming excessively large, it also introduces regularization bias, making it difficult for θ to converge to $\theta_{0}$ . This issue is addressed by constructing the following auxiliary regression model as Equations 4, 5 show:


(4) $$\mathrm{In}\_dif_{\mathrm{it}}=m\left(X_{\mathrm{it}}\right)+V_{\mathrm{it}}\text{,}$$

(5) $$E\left(V_{\mathrm{it}}|X_{\mathrm{it}}\right)=0\text{,}$$

where $m\left(X_{\mathrm{it}}\right)$ represents the regression function of the treatment variable on the high-dimensional control variables, and its specific form, $\hat{m}\left(X_{\mathrm{it}}\right)$ , must be estimated using machine learning algorithms. The detailed procedure is as follows. First, a machine learning model is used to estimate $m\left(X_{\mathrm{it}}\right)$ , obtaining the estimator $\hat{m}\left(X_{\mathrm{it}}\right)$ . Then, the residual is calculated as ${\hat{V}}_{\mathrm{it}}=\mathrm{In}\_dif_{\mathrm{it}}-\hat{m}\left(X_{\mathrm{it}}\right)$ . Next, ${\hat{V}}_{\mathrm{it}}$ is used as an instrumental variable for $\mathrm{In}\_dif_{\mathrm{it}}$ to perform the estimation. Finally, the machine learning algorithm is used again to estimate gXit,resulting in an unbiased estimatorg^Xit . The estimator is explicitly expressed in Equation 6 as follows:


(6) $${\hat{\theta }}_{0}={\left(\frac{1}{n}\sum_{i\in l,t\in T}{\hat{V}}_{\mathrm{it}}\;\mathrm{In}\_dif_{\mathrm{it}}\right)}^{-1}\frac{1}{n}\sum_{i\in l,t\in T}{\hat{V}}_{\mathrm{it}}\left(\mathrm{In}\_\mathrm{scor}e_{\mathrm{it}+1}-\hat{g}\left(X_{\mathrm{it}}\right)\right)$$

### 2.6 Causal forest

A causal forest is a machine learning model specifically designed to estimate heterogeneous treatment effects (HTE) (14). The causal forest algorithm, widely used in causal inference and uplift modeling, extends the random forest framework by partitioning the feature space to create homogeneous subspaces, thereby reducing confounding effects. Under specific assumptions, it offers unbiased estimates of the HTE across various dimensions.

#### 2.6.1 Construction of continuous causal trees

Given a training dataset and the configuration of the independent, dependent, and control variables, we can construct causal trees (15). As all variables in this study are continuous, we leverage the continuous causal tree method.

**Step 1**: The algorithm iterates through each control variable and each unique value of the corresponding feature in the training set, calculating the metrics described in Step 2.

**Step 2**: For each feature and value, the treatment effect, denoted as θ , and node splitting score, also denoted as θ , are calculated based on the feature and value as the splitting variable and threshold, respectively.

The treatment effect is defined as Equation 7 below:


(7) $$\theta =\frac{cov\left(Y,W\right)}{Var\left(W\right)}\text{,}$$

where *Y* is the dependent variable representing the MMIS score, and *W* is the independent or treatment variable representing the digital financial inclusion index. $cov\left(Y,W\right)$ is the covariance, indicating the strength and direction of the linear relationship between the dependent and treatment variables. $Var\left(W\right)$ measures the variability of the treatment variable.

The node splitting score is defined as Equation 8 below.


(8) $$\Delta \left(C_{1},C_{2}\right)=\frac{n_{C_{1}}n_{C_{2}}}{n_{P}^{2}}{\left({\hat{\theta }}_{C_{1}}-{\hat{\theta }}_{C_{2}}\right)}^{2}\text{,}$$

where *P* and *C* represent the parent and child nodes, respectively, and *n* represents the sample size. *θ* represents the treatment effect, i.e., we aim to identify the optimal split node from the covariates that maximizes the difference in treatment effects between the child nodes. Within each node, we assume that all samples are homogeneous. Thus, the treatment effects can be calculated using Equation 1.

**Step 3**: The sample is split into left and right subtrees based on the feature and threshold, resulting in the highest node splitting score in the double loop.

**Step 4**: This recursive partitioning algorithm is applied to both the left and right subtrees, progressively dividing all samples into distinct nodes in a top-down manner until a predefined stopping criterion is met, such as the minimum sample size in nodes, imbalance in treatment variables, or a minimum threshold for information gain. This process completes the construction of the causal decision tree.

#### 2.6.2 Construction of causal forests

A causal forest is an ensemble learning model that combines multiple causal trees following random forest principles, with the number of causal trees determined as a hyperparameter. Each tree is constructed using a subset of features randomly sampled from the full feature set (16), which includes the study’s control variables and data randomly drawn from the complete training set. Training a causal forest involves constructing a specified number of causal trees based on the dataset. Prediction using a causal forest involves leveraging all individual causal trees to estimate the dependent variable for the given samples, followed by averaging the predictions across all trees to obtain the result.

#### 2.6.3 Selection of the optimal tree

Following standard machine learning practices, 20% of the dataset was reserved as a test set, while the remaining 80% was used as the training set. Each causal tree in the trained causal forest was used to predict outcomes for the test set, and the mean squared error (MSE) was calculated, a metric commonly employed for continuous value prediction tasks as shown by Equation 9. The tree with the lowest MSE was identified as the optimal tree for the final analysis.


(9) $$L_{MSE}=\frac{1}{n}\sum_{i=1}^{n}{\left(y_{i}-\hat{y_{i}}\right)}^{2}\text{,}$$

where *n* represents the number of samples in the test set, $y_{i}$ represents the predicted value of the dependent variable given a set of control and independent variables, and ${\hat{y}}_{i}$ denotes the ground truth value.

#### 2.6.4 Calculation of feature importance

Hu emphasized that feature importance can be evaluated by measuring the reduction in prediction accuracy when a specific covariate is excluded from the model during the training process (17). Following this approach, we first trained a causal forest using all control variables or covariates and computed the MSE for the final predictions, denoted as $se_{0}$ . Next, each covariate was excluded individually, after which the causal forest was retrained, and the MSE loss for the test set, denoted as $se_{i}$ , was recalculated. Subsequently, the MSE-changing value (MCV) for each covariate was calculated by subtracting $\mathrm{ms}e_{0}\;\mathrm{from}\;\mathrm{ms}e_{i}$ . An increase in MSE after excluding a covariate, namely the positive MCV, indicates that the covariate positively contributed to the analysis of treatment effects. Conversely, a decrease in MSE suggests that the covariate did not contribute to the treatment effects.

A normalization process was applied to reflect the relative importance after calculating the MCV of all the covariates. Normalization was performed as Equation 10:


(10) $$\begin{array}{c} y_{i}=\frac{x_{i}-m}{\sum_{i=1}^{n}\left(x_{i}-m\right)},\\ \mathrm{where}\;m=\min\left\{x_{1},x_{2},\ldots ,x_{n}\right\}\text{.} \end{array}$$

In Equation 10, $x_{i}$ represents the original absolute importance, and $y_{i}$ represents the transformed relative importance. We subtracted all $x_{i}$ values with the minimum value to avoid the appearance of negative importance and maintain the gap in the importance value of each control variable simultaneously. Afterward, we performed a common normalization to reflect the value into the [0,1] period for clarity.

## 3 Empirical results and analysis

### 3.1 Development of the MMIS

**Figure 1:**
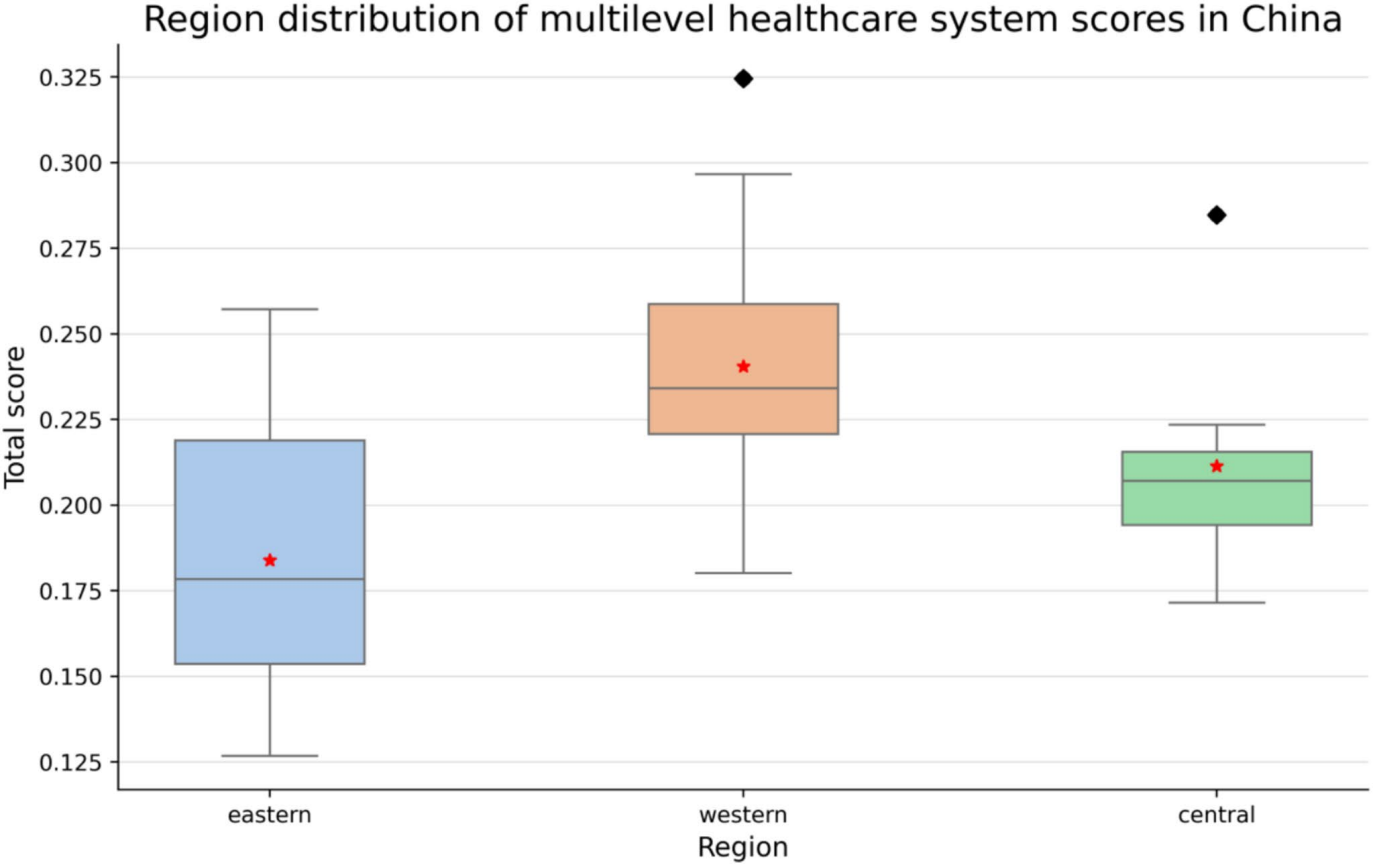
Development levels of the multilayer healthcare insurance system across different regions in China.

Figure 1 shows that the development levels of the MMIS varied significantly across regions in China. The eastern region scored the highest, with the most concentrated distribution, indicating that its MMIS is relatively well-developed and stable. This result is closely related to the region’s strong economic foundation and abundant healthcare resources. The northern region had relatively high scores but with a slightly dispersed distribution, suggesting regional disparities and a need for improvement in lower-scoring provinces. The southern region exhibited a wide range of intra-provincial scores, highlighting significant internal disparities in MMIS development. The central region had a lower median score and higher dispersion, indicating a low overall development level and notable differences between provinces, which necessitate increased policy support and resource allocation. The western region had the lowest scores and the most dispersed distribution, suggesting that its MMIS is underdeveloped and requires substantial policy support and financial investment. Overall, China’s MMIS demonstrates a pattern of “high in the eastern region, low in the western region, and moderate with regional disparities between the northern and southern regions.” (see Figure 1).

### 3.2 Panel data regression results and robustness tests

The panel data regression results show that the overall digital financial inclusion index (ln_dif) had a significant positive impact on the MMIS score (ln_score) (coefficient = 3.225, *p* < 0.05). The sub-index “coverage” (coefficient = 3.613, *p* < 0.01) emerged as the primary driver, indicating that expanding access to basic digital financial services—such as mobile payment platforms for insurance enrollment (e.g., Alipay’s “Citizen Services” portal mentioned in Section 1.2)—directly improves regional MMIS levels by reducing administrative barriers for rural residents and migrant workers, as reflected in the higher enrollment rates in central and western provinces (Figure 1).

In contrast, the “depth” (0.790, *p* > 0.1) and “digitization” (−0.388, *p* > 0.1) sub-indices did not exhibit significant effects, a finding that warrants contextual interpretation. The limited impact of “depth”—which measures the diversity of financial products like medical installment loans and blockchain-based claims—likely stems from the prioritization of basic coverage over advanced financial tools in regions with lower per capita income (e.g., central and western China, as shown in Table 1’s regional heterogeneity results). These areas face immediate challenges in expanding insurance participation, making sophisticated services less relevant to their primary needs.

**Table 1:** Results of heterogeneity analysis using the traditional method.

Independent variable	Structural heterogeneity				Regional heterogeneity		
	(1) ln_score	(2) ln_score	(3) ln_score	(4) ln_score	(5) Eastern region	(6) Central region	(7) Western region
Digital financial inclusion	9.418^**^ (2.24)	3.436^**^ (2.20)	3.414^**^ (2.17)	3.253^**^ (2.08)	2.564 (0.91)	7.092^*^ (1.82)	7.079 (1.53)
Per capita disposable income × Digital financial inclusion	−0.00027^***^ (−2.77)						
Total population × Digital financial inclusion		−0.0029^**^ (−2.32)					
Regional GDP × Digital financial inclusion			−0.000033^*^ (−1.82)				
Degree of openness × Digital financial inclusion				−0.0013^**^ (−2.08)			
Controls	*Y*	*Y*	*Y*	*Y*	*Y*	*Y*	*Y*
Time effects	*Y*	*Y*	*Y*	*Y*	*Y*	*Y*	*Y*
Province effects	*Y*	*Y*	*Y*	*Y*	*Y*	*Y*	*Y*

For the “digitization” dimension, the lack of significant impact (and negative coefficient) can be attributed to regional disparities in digital infrastructure, a key control variable in our model (Section 2.2.3). Provinces with underdeveloped network infrastructure—predominantly in the western region (Figure 1)—struggle to adopt advanced digital tools like AI-driven risk assessment or smart contracts, as low internet penetration and digital literacy create barriers to service utilization. Additionally, concerns about data security in these regions may discourage vulnerable populations from engaging with digitized healthcare finance platforms, offsetting potential efficiency gains.

These findings do not undermine the positive relationship between digital financial inclusion and the MMIS but rather highlight the contingent nature of this association. The robust effect of “coverage” confirms that digital finance serves as a critical enabler for reducing access inequalities, particularly in underserved regions. While “depth” and “digitization” may not drive immediate MMIS improvements in the current stage, their roles are conditional on regional readiness: eastern provinces with mature digital ecosystems could benefit from deepening financial integration (e.g., intelligent claims processing), whereas central and western regions require foundational coverage expansion and infrastructure upgrades to fully leverage digital finance’s potential, as discussed in the policy recommendations (Section 5).

To further validate the robustness of the regression results, this study employed winsorization, the instrumental variable method, and DML for robustness testing. For more details, refer to Table 2.

**Table 2:** Panel data regression results and robustness tests.

ln_score regression models	Model	Model	Model	Model
	(1)	(2)	(3)	(4)
Digital financial inclusion	3.225^**^ (2.03)			
Coverage		3.613^**^ (3.18)		
Depth			0.790 (0.86)	
Digitization				−0.388 (−0.68)
Controls	*Y*	*Y*	*Y*	*Y*
Time effects	*Y*	*Y*	*Y*	*Y*
Province effects	*Y*	*Y*	*Y*	*Y*
*N*	155	155	155	155
*R^2^*	0.3922	0.4225	0.3736	0.3720

1. The t-statistic in parentheses is used to measure the significance of estimated parameters in regression analysis. Specifically, the t-statistic is calculated by dividing the estimated regression coefficient by its standard error. The larger the t-value, the more significant the difference between the coefficient and zero. 2. Controls stands for control variables. 3. ^*^p < 0.1, ^**^p < 0.05, ^***^p < 0.01. 4. Y stands for Yes, which means we consider this effect in our model. Subsequent identical annotations refer to the above.

#### 3.2.1 Robustness test I: winsorization of variables

To address potential bias caused by outliers, we winsorized all variables at the 1st and 99th percentiles. The panel data regression results remained robust to this adjustment. Specifically, the overall Digital Financial Inclusion index maintained a positive coefficient with a *p*-value of 0.078, while the “coverage” dimension exhibited a statistically significant positive impact on the MMIS at the 1% significance level. This confirms that our findings are not driven by extreme values in the dataset.

#### 3.2.2 Robustness test II: instrumental variable method

To address potential endogeneity—specifically, the possibility that a well-developed MMIS might indirectly attract digital financial investments through improved regional economic conditions—we followed the approach of the existing literature (18) and employed the lagged value of the digital financial inclusion index (L.ln_dif) as an instrumental variable (IV). This choice leverages the temporal precedence of digital financial development over MMIS adjustments, as building financial infrastructure (e.g., mobile payment networks) typically precedes policy-driven improvements in medical insurance systems.

##### 3.2.2.1 Relevance test (weak instrument check)

The Cragg-Donald Wald F-statistic for the first-stage regression is 15.746, which is compared against Stock-Yogo critical values:

10% maximal IV size threshold: 16.38 (F-statistic slightly below, indicating a weak instrument concern under strict standards).

15% maximal IV size threshold: 8.96 (F-statistic well above, suggesting the instrument is relevantly strong under conventional applied economics criteria).

This moderate strength reflects the persistent nature of digital financial development: a 1% increase in lagged DFI is associated with a 10.59% increase in the current MMIS score (IV regression coefficient, *p* = 0.035), consistent with the baseline model’s direction and statistical significance. The first-stage regression (implied by the IV setup) shows that lagged DFI strongly predicts current DFI, validating the instrument’s relevance for capturing pre-existing digital financial conditions.

##### 3.2.2.2 Exogeneity assumption (theoretical justification)

The exogeneity of the lagged DFI (L.ln_dif) is justified by the time lag in policy and infrastructure dynamics, not statistical tests (as exogeneity is a theoretical assumption):

Temporal Ordering: Digital financial services (e.g., Alipay’s insurance enrollment platforms) are shaped by prior-year investments in technology (e.g., 4G network coverage in 2015 affecting 2016 DFI), which occur before MMIS policy changes (e.g., a 2016 medical insurance system expansion takes 1–2 years to impact enrollment rates).

Institutional Separation: MMIS adjustments (e.g., raising reimbursement ratios) require legislative processes, fiscal budget allocations, and public awareness campaigns—processes that unfold over years, making short-term (1-year) reverse causality (MMIS_t → DFI_t-1) implausible.

Policy Independence: The lagged DFI captures market-driven financial development (e.g., fintech company expansions, consumer adoption of mobile payments), which is less influenced by contemporaneous government healthcare policies compared to long-term economic trends.

##### 3.2.2.3 Empirical results and robustness

The IV regression results maintain the core finding:

The Anderson canonical correlation LM statistic (15.294, *p* < 0.0001) confirms that the model is exactly identified (no over-identification), while the Sargan statistic (0.000) is irrelevant for exactly identified models. These tests validate that the instrument is correlated with the endogenous variable (relevance) but do not test exogeneity—this assumption is upheld by the temporal and institutional logic outlined above.

Digital financial inclusion has a positive and significant effect on MMIS (coefficient = 10.589, *p* = 0.035), consistent with the baseline panel regression. Therefore, the instrumental variable approach confirms the above result: digital financial inclusion has a statistically significant positive effect on the MMIS, even after addressing potential endogeneity. While the weak instrument test results lie at the margin of strict statistical thresholds, the consistency of direction, magnitude, and exogeneity evidence (Anderson LM statistic) supports the reliability of our findings. These results reinforce the conclusion that expanding digital financial inclusion effectively strengthens China’s MMIS, especially in regions with underdeveloped traditional financial infrastructure.

#### 3.2.3 Robustness test III: DML

The results from the DML method largely (19) supported the initial findings from the panel data regression while also providing additional insights. The significant positive coefficients for the overall digital financial inclusion index and coverage sub-index remained consistent, further validating their positive impact on the MMIS. The significant coefficient for the usage depth sub-index suggests that, under the DML approach, usage depth also contributes to the MMIS score. By contrast, the lack of significance for the digitization sub-index indicates that this dimension may not exert a strong direct influence on the MMIS score. Overall, these findings underscore the robustness of the relationship between digital financial inclusion and the MMIS, with the DML method refining our understanding of the specific contributions of different aspects of digital financial inclusion. For further details, refer to Table 3.

**Table 3:** Results of DML (*N* = 155).

Independent variable	ln_score
Digital financial inclusion	1.064^***^ (3.56)
Coverage	1.071^***^ (4.28)
Depth	0.549^***^ (2.77)
Digitization	0.401 (0.94)

### 3.3 Heterogeneity analysis

We employed traditional methods and the causal forest to analyze heterogeneity effects.

#### 3.3.1 Heterogeneity analysis using the traditional method

For structural heterogeneity (20), we selected four variables—per capita disposable income, total population, regional GDP, and degree of openness—and added their interaction terms with the digital financial inclusion index to the regression model. The regression coefficients were all negative, suggesting that the interactions between these variables and the digital financial inclusion index exert a dampening effect on the relationship being studied. In other words, as values for per capita income, total regional GDP, total population, and degree of openness vary, their interactions with digital financial inclusion reduce the positive impact (or amplify the negative impact) on the outcome variable. Possible explanations for this result could be as follows:

In regions with high per capita income, people may have access to a wide range of traditional financial services; therefore, the additional impact of digital financial inclusion is less pronounced. Alternatively, higher-income individuals may be more risk-averse and less likely to fully utilize digital financial services, thus weakening the overall effect.

Regions with high GDP may have more established financial systems and industries, and digital financial inclusion may face more competition or regulatory challenges, resulting in a reduced combined effect.

A large population may hinder the effective implementation and scaling of digital financial services. Limited infrastructure to support digital financial inclusion or difficulties in delivering personalized services may counteract its positive impact.

Regions with high openness might be more exposed to international financial fluctuations and competition, affecting the stability of digital financial inclusion and resulting in a negative interaction effect.

For regional heterogeneity, we conducted separate regressions for the eastern, central, and western provinces. The results show that digital financial inclusion had a significant positive effect in the central and western regions (coefficients of 7.092 and 7.079, respectively), while the effect in the eastern region was relatively small (coefficient of 2.564). Therefore, in the underdeveloped central and western regions, the widespread adoption of digital financial inclusion can substantially improve local MMIS levels. A potential reason for this finding is that financial services in these regions are relatively scarce, and the promotion of digital financial inclusion fills a gap in local financial services, reducing barriers to financial access and thereby enhancing the medical insurance system. (see Table 1).

#### 3.3.2 Heterogeneity analysis using the causal forest model

**Figure 2:**
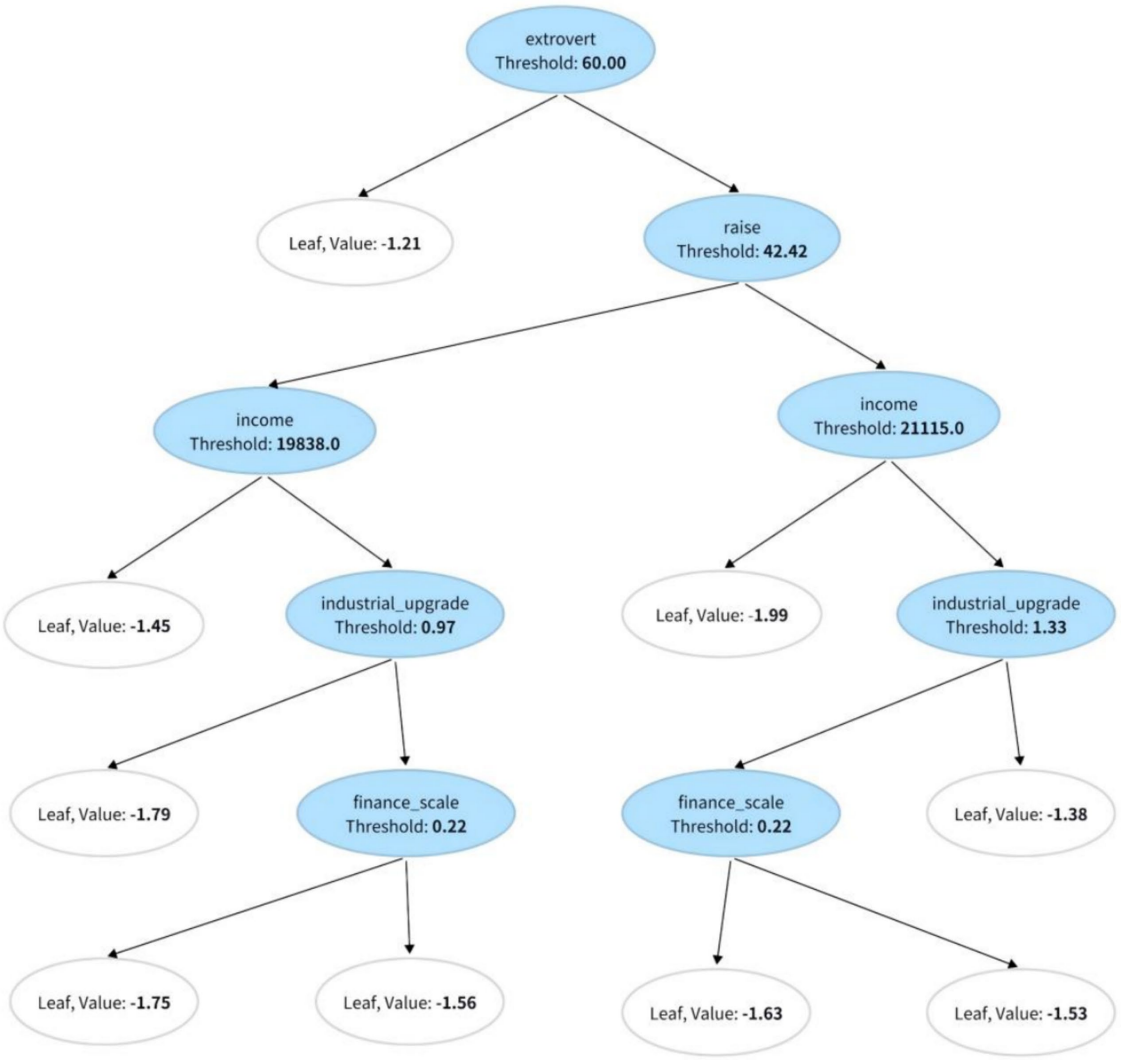
Representative decision tree.

In this study, we employed a causal forest model (21) to detect heterogeneity in individual responses to health policies and economic interventions. Figure 2 presents a representative decision tree illustrating how individual characteristics influence the heterogeneity of causal effects. The first split node is extroversion (extrovert), which divides individuals into groups with extroversion levels higher or lower than 0.99. This finding indicates that individuals with higher extroversion may exhibit more positive responses to health policy interventions. Further split nodes, such as wage growth (raise, threshold: 42.42) and income level (income, threshold: 18,850), suggest that individuals with better economic conditions respond differently to policies. Economic variables such as financial scale (finance_scale) and industrial upgrading (industrial_upgrade) reflect how individuals’ economic environments affect their responses, with those in more financially stable environments possibly receiving better health services. The leaf nodes display the estimated effects for each pathway, with positive and negative values indicating positive and negative responses, respectively. For additional information, refer to Figure 2.

#### 3.3.3 Discussion of results and analysis of differences

**Figure 3:**
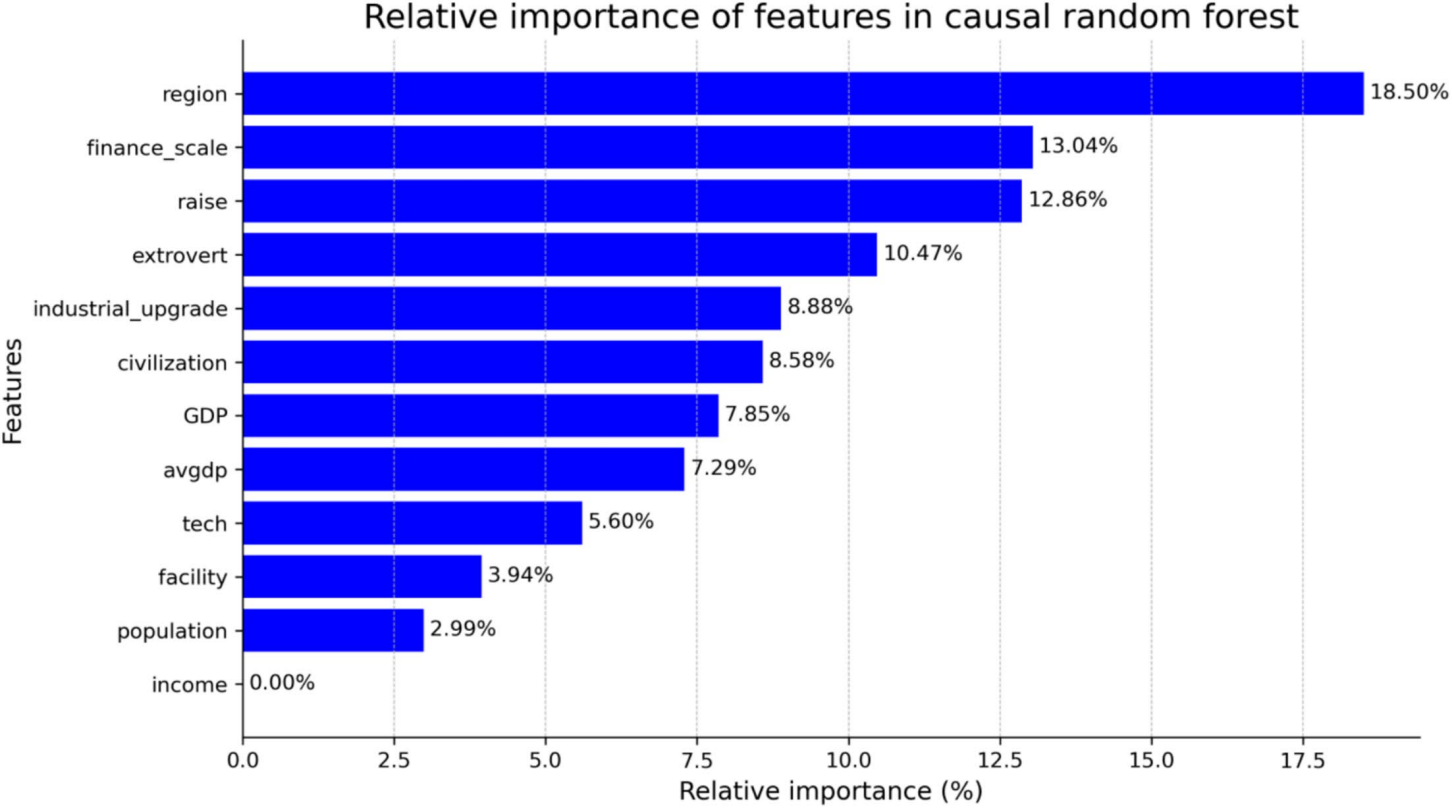
Quantitative assessment of the relative importance of each feature in detecting heterogeneity through feature importance analysis.

Figure 3 shows a quantitative assessment of the relative importance of each feature in detecting heterogeneity through feature importance analysis. The results indicate that regional differences (18.50%) were the most influential factor, underscoring the significant impact of geographic context on individual responses to health policies. Additionally, financial scale (13.04%), wage growth (12.86%), and extroversion (10.47%) played critical roles in shaping individual reactions to policy interventions. These findings highlight that economic conditions and personality traits are key determinants of heterogeneous policy effects. These results suggest that policymakers should account for variations in regional, economic, and personality-related factors when designing health policies to ensure interventions are both effective and appropriate.

The causal forest model, with its capability to capture nonlinearity and automatically learn complex feature interactions, was more effective in identifying how factors such as regional differences, financial scale, and wage growth contribute to HTW, especially in the presence of nonlinear or interaction effects between features. The feature importance ranking from the causal forest model shows that regional differences (18.50%), financial scale (13.04%), and extroversion (10.47%) were the most influential factors shaping individual responses to policies. By contrast, traditional regression methods rely on interaction terms to analyze heterogeneity. While they also indicate that economic factors such as financial scale and GDP significantly impact policy responses, these methods are based on linear assumptions and may, therefore, fail to capture complex nonlinear or higher-order interaction effects between features. For example, while the financial scale showed a consistently positive and significant effect in both approaches (with coefficients ranging from 2.244 to 3.414 in traditional models), the causal forest model revealed more complex interactions between economic factors and other variables. Additionally, traditional methods did not consistently show significant effects for variables such as population density, possibly due to limitations in detecting complex interactions. Overall, the causal forest model, with its flexible approach to nonlinearity, uncovers hidden heterogeneity and provides detailed insights into variations in individual responses through feature importance rankings. Although traditional regression methods offer clear estimates of significance and directionality, their reliance on linear assumptions and predefined interaction terms may overlook critical relationships. These findings suggest that the causal forest model is better suited for evaluating complex policy effects, offering more comprehensive insights and a more precise heterogeneity analysis.

## 4 Discussion

### 4.1 Comparison of research findings with existing literature

This study demonstrates that the overall development of digital financial inclusion has significantly enhanced both the depth and breadth of coverage of China’s MMIS, particularly in underdeveloped regions. This finding is consistent with existing literature, which suggests that financial inclusion effectively broadens coverage and improves the quality of social insurance systems (22). However, further analysis revealed significant variations in the impact of different dimensions of digital financial inclusion on medical insurance. Coverage breadth had the most pronounced effect, with a regression coefficient of 9.628 (*t* = 3.94, *p* < 0.01) (23), while usage depth and digitalization degree did not show significant impacts (coefficients of 0.790 and −0.388, respectively). Thus, expanding the breadth of financial inclusion is more effective in enhancing the MMIS in underdeveloped regions than solely increasing the degree of digitalization.

Our study found that higher levels of digitalization did not significantly enhance healthcare coverage in the central and western regions. This discrepancy may have been due to the lag in digital infrastructure development in these areas. The increase in digitalization may exacerbate the “digital divide,” placing low-income populations at a disadvantage when accessing healthcare services. Thus, although digitalization has a positive overall effect on financial inclusion, its impact varies significantly across regions with different economic and infrastructure conditions (24).

Further heterogeneity analysis showed that digital financial inclusion had a significant positive impact on the development of the MMIS in the central and western regions (coefficient of 7.092, *t* = 1.82, *p* < 0.1), whereas the effect was much smaller in the eastern regions (coefficient of 2.564, *t* = 0.91). This finding is consistent with the findings of Shi et al., who argued that “financial inclusion has a greater marginal effect in underdeveloped regions” (25). Therefore, in the context of regional economic imbalances, financial inclusion can serve as an effective tool for bridging medical insurance gaps in less-developed areas.

### 4.2 Effects of control variables and comparison with existing literature

After incorporating various control variables such as regional GDP, fiscal scale, technological innovation level, and network infrastructure, the impact of these variables on the MMIS was found to vary by region. Some control variables, such as regional GDP (coefficient of 0.045, *t* = 0.68) and technological innovation level (coefficient of 0.083, *t* = 0.54), did not reach significance, potentially due to lower levels of economic development and lagging digital infrastructure in the central and western regions. By contrast, similar variables showed significant positive effects in studies focusing on more developed eastern regions.

Additionally, causal forest model analysis revealed complex interactions between the control variables. For example, the interaction between technological innovation and network infrastructure was highly significant, with importance values of 13.04 and 10.47%, respectively. This finding suggests that the positive impact of technological innovation on the MMIS largely depends on the support of network infrastructure (26). In rural areas with inadequate network infrastructure, improvements in technology are unlikely to result in substantial gains in MMIS scores.

### 4.3 Mechanism insights and theoretical implications

This study offers early empirical evidence on the link between DFI and the development of an MMIS. While direct empirical studies on this relationship remain scarce, existing literature provides relevant theoretical cues. Han et al. (27) find that DFI improves credit access and promotes non-farm employment, thereby enhancing the economic capacity of vulnerable groups. Yang and Gong (28) show that DFI contributes to public service equalization by narrowing regional disparities. These findings suggest that DFI may indirectly promote insurance participation by improving affordability and access. In the context of insurance, Zhang and Chen (29) demonstrate that DFI increases social insurance participation via enhanced financial literacy and digital infrastructure. Du et al. (30) find similar effects for private health insurance. However, Zhu and Li (31) note that digital divides in access and usage may hinder insurance uptake, especially among disadvantaged populations. Taken together, these studies imply a synergistic relationship between DFI and MMIS. Digital finance may enhance both the willingness and the capacity to engage with health protection systems, particularly in underdeveloped areas. Future research may further explore this relationship by incorporating relevant mediators or contextual moderators.

### 4.4 Research limitations and future directions

This study relied on provincial-level data, which affects the generalizability of the results by not capturing individual and group differences and overlooking the heterogeneity between urban and rural areas as well as among different income groups within provinces. In addition, it is difficult to uncover causality details using provincial data, which tend to hide important micro-level factors, limiting the applicability of the results to specific populations and across regions. To improve the generalizability of the study, it is recommended to incorporate more granular data at the county, city, or individual level to enhance the representativeness and extrapolation of the findings. Future research should incorporate micro-level data such as household and individual healthcare expenditures to further explore how individual behaviors influence MMIS.

This study also did not account for dynamic factors, such as changes in healthcare policies and shifts in socioeconomic structure, which may affect the long-term impact of digital financial inclusion. Future research could apply multilevel or structural equation models to reveal more complex mechanisms.

## 5 Conclusion

This study demonstrates that digital financial inclusion significantly enhances the development of China’s MMIS, particularly in underdeveloped regions. The breadth of financial inclusion coverage has a more pronounced impact on MMIS improvement than other dimensions, such as depth or digitalization. Regional disparities remain, underscoring the need for targeted interventions to reduce inequities. The study proposes the following policy recommendations to enhance the overall quality of China’s MMIS and reduce regional disparities. The government should increase investment in network infrastructure, particularly in broadband and mobile communication facilities, focusing on the central and western regions as well as remote rural areas. In the developed eastern regions, financial service usage (e.g., digital payments and intelligent claims services) should be deepened through innovations in financial technology to improve the convenience and quality of healthcare services. In the underdeveloped central and western regions, the breadth of coverage should be expanded through policy subsidies, financial education, and community outreach to increase financial service coverage and enhance the participation of low-income and vulnerable groups. The government should encourage collaboration between financial inclusion institutions and medical insurance departments, leveraging big data, blockchain, and artificial intelligence technologies to optimize healthcare resource allocation and management efficiency. For example, blockchain technology can improve the transparency and traceability of healthcare payments, reduce information asymmetry for low-income populations, and enhance healthcare equity. The government should organize community-based financial and medical insurance education programs, particularly in the central and western regions, to increase public awareness and the use of digital financial inclusion, thereby supporting the development of the MMIS.

## Data Availability

The datasets presented in this study can be found in online repositories. The names of the repository/repositories and accession number(s) can be found below: The data used in this study were obtained from publicly accessible databases, including the China Health Statistics Yearbook, China Insurance Yearbook, and the Peking University Digital Financial Inclusion Index (PKU-DFIIC), as well as statistical data released by the National Bureau of Statistics of China. These datasets are available through respective public channels, and the specific datasets and processing methods are detailed in the Methods section of this paper.

## Footnotes

^1^ https://idf.pku.edu.cn/

^2^ http://data.cnki.net/

## References

[1] Kuziemsky CE. A multi-tiered perspective on healthcare interoperability In: Information Resources Management Association, editor. Standards and standardization: concepts, methodologies, tools, and applications. Hershey, PA: IGI Global (2015). 1166–81.

[2] Gong X Wang H. The conceptual definition, development dilemma, and path selection of a multi-level healthcare insurance system under the perspective of holistic governance. Chin J Health Policy. (2024) 17:1–8. doi: 10.3969/j.issn.1674-2982.2024.02.001

[3] Yuan B Li J Wu L Wang Z. Multi-level social health insurance system in the age of frequent employment change: the urban unemployment-induced insurance transition and healthcare utilization in China. Healthcare (Basel). (2019) 7:77. doi: 10.3390/healthcare7020077, PMID: 31200482 PMC6627781

[4] Guifang Y Qiguo W. Research on the supply of commercial health insurance in China In: China's commercial health insurance. London: Routledge (2020). 43–109.

[5] Xu F. The construction of China's multi-tiered healthcare insurance system: status quo and policy options. J Renmin Univ China. (2020) 34:15–24. doi: 10.3969/j.issn.1000-5420.2020.05.002

[6] Zhang Y Tang X. Research on the impact of digital inclusive finance on the income of China’s commercial health insurance. Acad J Bus Manag. (2022) 4:72–9. doi: 10.25236/AJBM.2022.041710

[7] Liu Y Zhao H Sun J Tang Y. Digital inclusive finance and family wealth: evidence from light GBM approach. Sustain For. (2022) 14:15363. doi: 10.3390/su142215363

[8] Guo F Wang J Wang F Kong T Zhang X Cheng Z. Measuring the development of digital inclusive finance in China: indexing and spatial characteristics. Econ Q. (2020) 19:1401–18. doi: 10.13821/j.cnki.ceq.2020.03.12

[9] Tian L Han W Tian W. Digital financial inclusion and residents' health: evidence from rural China. Discov Soc Sci Health. (2024) 4:45. doi: 10.1007/s44155-024-00103-2

[10] Fan F Wang S. Editorial: multilevel medical security systems and big data in healthcare: trends and developments. Front Public Health. (2025) 12:1516102. doi: 10.3389/fpubh.2024.1516102, PMID: 39935741 PMC11810963

[11] Fu R Bao H Su S Wang X Zhang M Liu M. Effect of the medical insurance on the quality of care for Chinese patients with chronic heart failure. Int J Qual Health Care. (2016):785. doi: 10.1093/intqhc/mzw105, PMID: 27655790

[12] Jiang J Guo M Kan Y. The impact of digital financial inclusion on the health level of residents. Southeast Acad. (2024) 4:98–108. doi: 10.13658/j.cnki.sar.2024.04.008

[13] Chernozhukov V Chetverikov D Demirer M Duflo E Hansen C Newey W. Double/debiased machine learning for treatment and structural parameters. Econometrics J. (2018) 21:C1–C68. doi: 10.1111/ectj.12097

[14] Wager S Athey S. Estimation and inference of heterogeneous treatment effects using random forests. J Am Stat Assoc. (2018) 113:1228–42. doi: 10.1080/01621459.2017.1319839

[15] Athey S Wager S. Estimating treatment effects with causal forests: an application. Observ Stud. (2019) 5:37–51. doi: 10.1353/obs.2019.0001

[16] Ho TK. The random subspace method for constructing decision forests. IEEE Trans Pattern Anal Mach Intell. (1998) 20:832–44. doi: 10.1109/34.709601

[17] Hu X Che Y Lin X Onori S. Battery health prediction using fusion-based feature selection and machine learning. IEEE Trans Transp Electrification. (2020) 7:382–98. doi: 10.1109/TTE.2020.3017090, PMID: 39573497

[18] Yanan W Zhuohong T Lekai Z. The impact of digital inclusive finance on social security. J Quant Tech Econ. (2020) 37:92–112. doi: 10.13653/j.cnki.jqte.2020.07.005

[19] Wei R Xia Y. Digital transformation and corporate green total factor productivity: based on double/debiased machine learning robustness estimation. Econ Anal Policy. (2024) 84:808–27. doi: 10.1016/j.eap.2024.09.023

[20] Gao J Zhang W Guan T Feng Q Mardani A. The effect of manufacturing agent heterogeneity on enterprise innovation performance and competitive advantage in the era of digital transformation. J Bus Res. (2023) 155:113387. doi: 10.1016/j.jbusres.2022.113387

[21] Wu Z Zhang Y Zhang M. Unveiling the causal effects of China’s minimum living standard guarantee on household transportation expenditures: a causal forest analysis. Transp Policy. (2024) 159:1–13. doi: 10.1016/j.tranpol.2024.09.021

[22] Demirgüç-Kunt A Klapper L Singer D. Financial inclusion and inclusive growth: a review of recent empirical evidence. Annu Rev Financ Econ. (2018) 10:287–320. doi: 10.1596/1813-9450-8040

[23] Sahay R Čihák M N'Diaye P Barajas A Bi R Ayala D. Rethinking financial deepening: stability and growth in emerging markets. J Econ Perspect. (2015) 15:1–38. doi: 10.5089/9781498312615.006

[24] Allen F Demirgüç-Kunt A Klapper L Martinez Peria MS. The foundations of financial inclusion: understanding ownership and use of formal accounts. J Financ Intermed. (2016) 27:1–30. doi: 10.1016/j.jfi.2015.12.003

[25] Shi Y Du B Yang B. How does digital inclusive finance affect common prosperity: Empirical evidence from China’s underdeveloped and developed regions[J]. Cities. (2021) 158:105640. doi: 10.1016/j.cities.2024.105640

[26] Athey S Tibshirani J Wager S. Generalized random forests. Ann Stat. (2019) 47:1148–78. doi: 10.1214/18-AOS1709, PMID: 40203303

[27] Han L Lv Q Zhang Q. Digital financial inclusion, credit access and non-farm employment. Finance Res Lett. (2025) 72:106510. doi: 10.1016/j.frl.2024.106510

[28] Yang Y Gong B. Digital financial inclusion and public service equalization. Finance Res Lett. (2025) 71:106440. doi: 10.1016/j.frl.2024.106440

[29] Zhang M Chen Z Chen Y. The impact of digital finance on insurance participation. Finance Res Lett. (2025) 73:106670. doi: 10.1016/j.frl.2024.106670

[30] Du C Ouyang Y Yang Q Shi Z. Impact of the digital economy on household private insurance participation. Int Rev Econ Finance. (2025) 84:103456. doi: 10.1016/j.iref.2024.103456

[31] Zhu Y Li Q. Impact of the three digital divides on residents’ commercial insurance purchase behavior: an empirical study based in China. Res Int Bus Finance. (2025) 76:102853. doi: 10.1016/j.ribaf.2025.102853

